# APC mutations disrupt β-catenin destruction complex condensates organized by Axin phase separation

**DOI:** 10.1007/s00018-023-05068-0

**Published:** 2024-01-27

**Authors:** Dan Zhang, Qi-Qi Ni, Shu-Yang Wang, Wen-Feng He, Ze-Xuan Hong, Hui-Ye Liu, Xiao-Hong Chen, Li-Jie Chen, Fang-Yi Han, Ling-Jie Zhang, Xiao-ming Li, Yan-qing Ding, Hong-li Jiao, Ya-ping Ye

**Affiliations:** 1 Department of Pathology, Nanfang Hospital, Southern Medical University, Guangzhou, Guangdong China; 2 Department of Pathology, School of Basic Medical Sciences, Southern Medical University, Guangzhou, Guangdong China; 3 Guangdong Province Key Laboratory of Molecular Tumor Pathology, Guangzhou, Guangdong China; 4 Jinfeng Laboratory, Chongqing, China; 5 Department of Pathology, The People’s Hospital of Baoan Shenzhen, Shenzhen, Guangdong China

**Keywords:** Phase separation, Destruction complex, Wnt/β-catenin signaling pathway, Nuclear transport, APC mutations, Axin, Colorectal cancer

## Abstract

The Wnt/β-catenin pathway is critical to maintaining cell fate decisions. Recent study showed that liquid–liquid-phase separation (LLPS) of Axin organized the β-catenin destruction complex condensates in a normal cellular state. Mutations inactivating the APC gene are found in approximately 80% of all human colorectal cancer (CRC). However, the molecular mechanism of the formation of β-catenin destruction complex condensates organized by Axin phase separation and how APC mutations impact the condensates are still unclear. Here, we report that the β-catenin destruction complex, which is constructed by Axin, was assembled condensates via a phase separation process in CRC cells. The key role of wild-type APC is to stabilize destruction complex condensates. Surprisingly, truncated APC did not affect the formation of condensates, and GSK 3β and CK1α were unsuccessfully recruited, preventing β-catenin phosphorylation and resulting in accumulation in the cytoplasm of CRCs. Besides, we propose that the phase separation ability of Axin participates in the nucleus translocation of β-catenin and be incorporated and concentrated into transcriptional condensates, affecting the transcriptional activity of Wnt signaling pathway.

**Supplementary Information:**

The online version contains supplementary material available at 10.1007/s00018-023-05068-0.

## Introduction

The Wnt pathway plays crucial role during embryonic development, and aberration of this pathway is involved in various human malignant tumors [1]. Approximately 90% of colorectal cancer (CRC) cases display permanent activation of the Wnt signaling pathway [2]. The destruction complex is a dynamic multiprotein assembly that targets the critical Wnt-effector β-catenin proteins for proteasomal degradation [3]. Once the destruction complex is disassembled, β-catenin is released and translocated into the nucleus, activating the transcription of Wnt-responsive genes [4]. Researchers have speculated that APC and Axin may undergo phase separation in cells [3, 5], and recently, Chen et al. showed that liquid–liquid-phase separation (LLPS) of Axin organized the β-catenin destruction complex condensates under normal cellular conditions [6]. However, the mechanism of the formation of β-catenin destruction complex condensates that are organized by Axin phase separation is largely unknown. Truncations of the tumor suppressor APC are found in more than 80% of CRC cases and induce aberrant Wnt signaling that drives CRC [7, 8]. Nevertheless, whether APC mutations impact the destruction complex condensates organization is unclear during CRC initiation and progression.

Phase separation can explain the self-assembly and organization of membraneless bodies in living cells [9, 10]. Recent studies have shown that disease-related mutations and aberrant chromatin structure disrupt phase separation to promote tumorigenesis [11–13]. Axin and APC, as scaffold proteins of the β-catenin destruction complex, are largely unstructured multidomain proteins that include long intrinsically disordered regions (IDRs), which mediate weak multivalent intermolecular interactions [14–16]. The structural characteristics of the Axin and APC proteins suggest that phase separation might play a crucial role in the establishment of β-catenin destruction complex condensates [17–19]. Phase-separated Axin condensates recruit other components of the destruction complex, GSK3β, CK1α, and β-catenin, facilitating β-catenin phosphorylation by GSK3β [6]. In addition, APC is required for puncta accumulation in vivo, and puncta localization and function are regulated by Wnt signaling [17, 20]. Interestingly, truncated APC proteins can bind β-catenin but not degrade β-catenin [21]. It is necessary to probe how truncated APC perturbs the formation of β-catenin-destroying complexes and the components of destruction complex condensates in CRC.

Upon activation of the Wnt pathway, β-catenin localizes to the nucleus and interacts with the TCF/LEF-1 transcription factor, triggering activation of downstream genes. Despite extensive investigations, the mechanism underlying β-catenin nuclear translocation is still unclear. For β-catenin, an 86 kDa protein, nuclear import rather than transport with nuclear import factors via the classic nuclear localization signal (NLS) was observed [22], and interactions with FG-rich nucleoporins [23] imported into the nucleus through direct binding to the nuclear pore machinery formed via LLPS were observed. Besides, Axin was reported to shuttle between the cytoplasm and the nucleus, regulating the nuclear–cytoplasmic distribution of β-catenin [24], which suggests that Axin could be involved in the nuclear transport of β-catenin and could perform certain functions related to β-catenin in the nucleus. Recent evidence has shown that distinct hydrophobic “patches” in the N- and C-tails of β-catenin contribute to nuclear transport [25], and β-catenin is incorporated into Mediator condensates at super-enhancers and target gene activation, a process mediated by the IDRs of β-catenin [26]. However, the mechanism underlying the nucleus translocation and transcription of β-catenin is still largely unclear.

Here, we report that the mechanism by which APC mutations disrupt β-catenin destruction complex condensates is the failure of GSK 3β and CK1α to be recruited into droplets. We also provide a hypothesis that accumulated β-catenin in the cytosol is carried by Axin to enter the nucleus, as is incorporated into transcriptional condensates resulting in pathway activation.

## Materials and methods

### Mice

Wild-type and APC^min/+^ mice were housed under specific pathogen-free (SPF) conditions at the Southern Medical University of China. All mouse experiments were approved by the Institutional Animal Care and Use Committee and were in strict accordance with good veterinary practice as defined by the Southern Medical University Laboratory Animal Center.

### Clinical tissue specimens

For immunohistochemical and immunofluorescence studies, CRC tissues and matched normal tissues were collected by surgical resection from patients with primary colorectal adenocarcinoma at Southern Hospital of Southern Medical University (Guangzhou, China), and none of them received radiotherapy or chemotherapy before surgical removal. The diagnosis and staging of CRC or normal intestinal epithelial tissue (distant from the tumor) was verified by independent pathologists. All procedures were performed with the approval of the Southern Medical University Review Board.

### Live cell imaging

Live cell imaging experiments were performed on an LSM880 confocal microscope (Zeiss, 63× oil objective, NA 1.4, 1 Airy Unit) equipped with an incubation chamber and a heated stage at 37 °C.

For the time-lapse imaging experiment, puncta in live cells that were stimulated with continuous 561 nm laser scanning every 3 s for 3 h were visualized.

The FRAP assay was conducted using the FRAP module. The prebleaching image was taken with 2% of the maximum intensity of the 488 nm laser, and the region of interest (ROI) was bleached with 100% laser power. The time-lapse image was taken simultaneously. Recovery data were background corrected and normalized to the ROI intensity prior to bleaching. A reference ROI outside the bleached area was processed in the same way. GraphPad Prism was used to plot and analyze the FRAP results.

For 1,6-HD treatment, CRC cells were treated with 6% or 0% 1,6-HD (Macklin, 629–11-8) in 1 mL of cell culture media for 1 min at 37 °C. After treatment, the cells were washed with PBS, fixed in 4% paraformaldehyde (PFA) (Sigma-Aldrich, P6148) for 10 min, and stored at 4 °C until processing for immunofluorescence and microscopy.

For “OptoDroplet” system, Cry2 light-mediated phase separation, the constructed Cry2-mcherry-β-catenin plasmid was transfected to the HEK-293 T β-catenin KO1 monoclonal cell line, which had knocked out β-catenin. After 2 days, the confocal dishes were placed under a confocal microscope and the procedure was set: activated using a 488 nm laser for 1 s with an activation interval of 5 s, and observed with a 561 nm laser at activation interval, cell status was recorded simultaneously using Time series of LSM880 with Airyscan, and the entire process lasted 15 min.

### Protein purification and in vitro droplet formation assays

For protein expression, plasmids were transformed into *Escherichia coli* BL21 (TransGen Biotech, CD901-02) cells grown in LB media with 1000 μg/mL ampicillin. After 16 h of expression under 0.5 mM IPTG (BioFroxx, 1122GR005) at 20 °C, the cell pellets were collected, resuspended, and lysed in lysis buffer (5 μg/mL PMSF, 10 μg/mL Triton X-100). This denatured suspension was clarified by centrifugation at 12,000 × g for 10 min at 4 °C and sonicated.

Supernatants containing fusion proteins were loaded onto a His GraviTrap column (ThermoFisher, 88,221) or Glutathione Agarose (ThermoFisher, 1600). The eluted proteins were then concentrated, and the protein concentration was measured via a BCA assay and absorbance.

All liquid droplet formation assays were performed in 150 mM KCl (unless specified), 5 mM MgCl2, 10 mM cAMP (as indicated), 20 mM HEPES, pH 7.0, 1 mM EGTA, 1 mM DTT, 0.5 mM ATP, and 100 mg/ml polyethylene glycol 8000 (unless specified). Purified proteins were incubated at different stoichiometries and at various concentrations at room temperature for 1 h and imaged under DIC and/or fluorescence microscopy.

### Statistical analysis

All statistical analyses were performed with GraphPad Prism 9 and SPSS version 20.0. Quantitative data are presented as the mean ± SEM. Statistical analyses included Student’s *t* test, Wilcoxon-Mann–Whitney test, Chi-square test for contingency tables, or one-way ANOVA. Survival curves were plotted by the Kaplan–Meier method and compared using the log-rank test. *P* < 0.05 was considered significant.

## Results

### β-Catenin destruction complex constructed by Axin were assembled condensates via a phase separation process

**Fig. 1:**
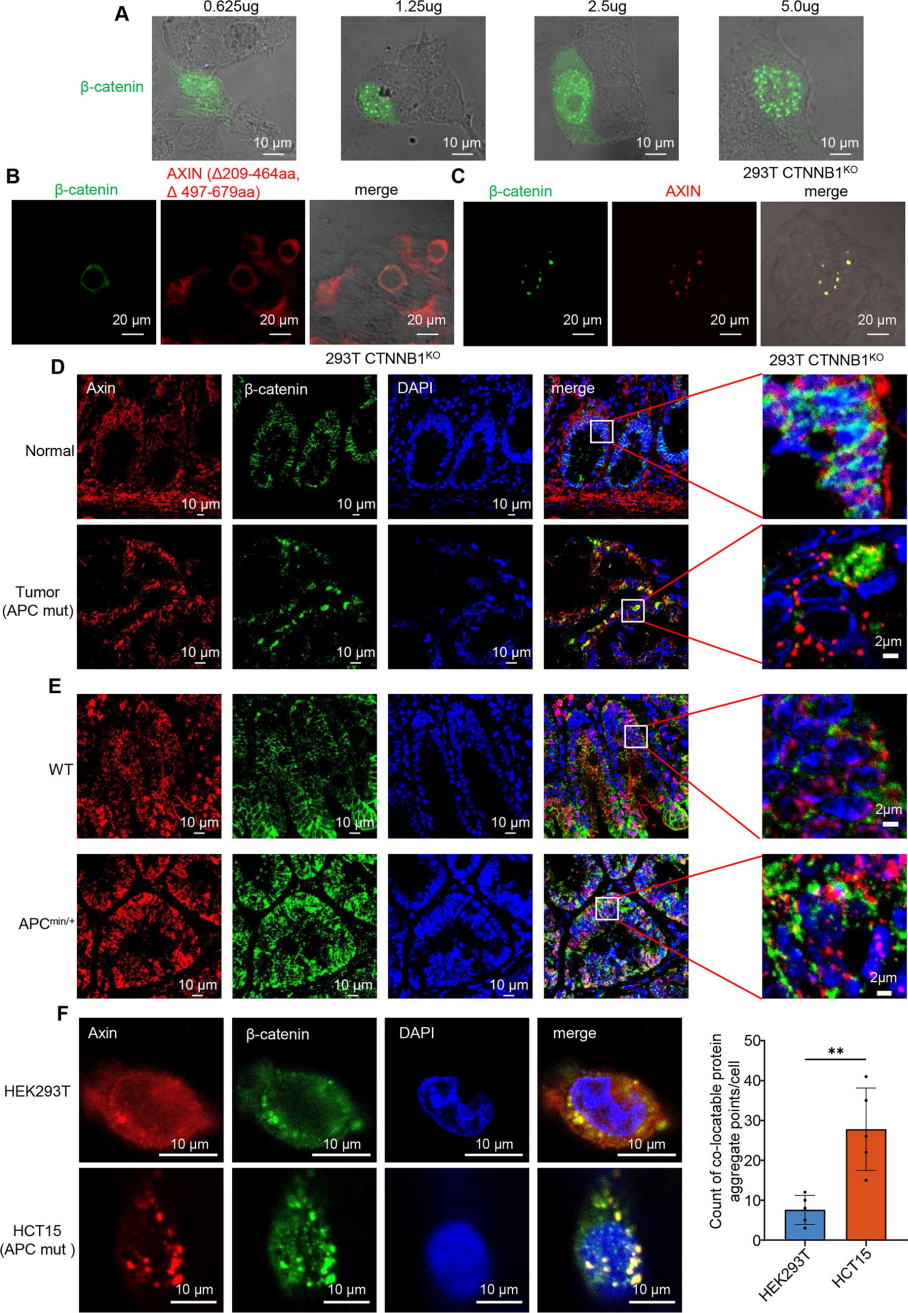
The formation of destruction complex condensates depends on the β-catenin accumulation of high concentrations in APC wild-type cells. **A** Representative live-cell images of β-catenin puncta formation after transiently expressing β-catenin at different protein concentrations in CTNNB1 KO HEK293T cells. **B**–**C** Representative live-cell images of β-catenin (green) and Axin (red) expression after transfection of β-catenin and mutant Axin (Δ209-464aa, Δ497-679aa) **B** or β-catenin and WT Axin **C** in CTNNB1 KO HEK293T cells. **D**–**E** Immunofluorescent staining of Axin (red), β-catenin (green), and nuclear (DAPI, blue) in FFPE tissues from CRC patients **D** or adjacent normal intestinal epithelial tissues, and APC^Min/+^ mouse adenocarcinoma tissues **E** or intestinal epithelial tissue of wild-type mice. **F** Left: immunofluorescent staining of Axin (red), β-catenin (green), and nuclear (DAPI, blue) in HCT15 cells. Right: quantitative determination of co-locatable protein aggregate points in 5 cells

Axin is the central scaffold of the destruction complex which directly interacts with all other core components of the complex [27, 28]. It has been reported that Axin protein forms droplets with liquid-like properties under normal physiological conditions [6]. Our results suggested that Axin IDRs (aa 209–679) are necessary and, to a certain degree, enough for Axin LLPS in CRCs (Figs. S1-2). To further explore how β-catenin is incorporated into the condensate formed by Axin, we generated β-catenin knockout (KO) HEK293T cells and Axin KO SW480 cells (Figs. S3A–B). The reporter assay showed EGFP-tagged β-catenin formed punctate structures in β-catenin KO HEK293T cells in a concentration-dependent manner (Fig. 1A). Besides, the "OptoDroplet" system [29], which uses light to activate IDR-mediated phase transitions in living cells, was applied for the study of β-catenin condensed phases. The result suggested that β-catenin undergone light-activated phase separation, forming liquid optodroplets (Fig. S3C). However, mutants lacking IDR (aa 209–464 and aa 497–679) but retaining the interaction region (aa 465–496) [30] between Axin and β-catenin failed to form puncta (Fig. 1B), while re-expression of Axin could form puncta containing β-catenin protein in β-catenin KO HEK293T cells (Fig. 1C). These results indicated that the droplet properties of the β-catenin destruction complex were determined by Axin IDR region.

We further elucidated the formation of β-catenin degradation complex in colorectal cancer cells. In the Axin KO SW480 cells, pScarlet [31] -tagged Axin formed punctate structures (Fig. S3D). Meanwhile, we used CRISPR/Cas9 to generate homozygous N-terminal insertions of the fluorescence protein pScarlet [31] in Axin loci in CRC cells. We validated the presence of Axin puncta by live-cell imaging of CRC cells with or without the APC mutation (Fig. S3E). Moreover, we transiently expressed the Axin protein with an N-terminal pScarlet tag in CRC cells, and time-lapse imaging revealed that these puncta were highly dynamic and formed larger droplets (Fig. S3F and Movie S1). Fluorescence recovery after photobleaching (FRAP) displayed the liquid–liquid-like properties of Axin droplets through rapid fluorescence recovery (Fig. S3G). Further, we found that Axin showed greater aggregates distribution in paraffin-embedded (FFPE) tissues from CRC patients than adjacent normal intestinal epithelial cells by immunofluorescence (IF), and the expression of β-catenin mostly colocalized with Axin (Fig. 1D). Similar results were observed in APC^Min/+^ mice [32] with induced adenoma carrying a loss-of-function germinal mutation in the APC gene (Fig. 1E). Additionally, we confirmed that endogenous β-catenin and Axin colocalized to more punctate structures in CRCs cells compared to normal cells (Fig. 1F). Together, these results revealed that β-catenin destruction complex constructed by Axin was assembled condensates via a phase separation process.

### The phase separation ability of APC can enhance the properties of β-catenin destruction complex condensates

APC is another complex multidomain scaffolding protein that is predicted to contain large IDRs (Figs. S4A–B). It was reported that APC is required to initiate and stabilize the formation of functional β-catenin destruction complexes [3, 6, 33]. And we investigated whether APC is involved in the β-catenin destruction complex condensates. IF demonstrated that the endogenous expression of APC showed a punctate pattern partially in human CRC cells regardless of the mutation of APC (Fig. S4C). Based on the predicted IDR (Fig. S4D), we selected the longest IDR starting at amino acid 2157 and ending at amino acid 2653 and constructed truncation mutants named APC-IDR. Droplets were discovered when APC-IDR was expressed independently in CRC cells (Fig. S4E), and the APC-IDR proteins were fully recruited to form colocalized puncta organized by Axin proteins in CRC cells and normal HEK293T cells (Fig. S4F). Moreover, Axin-IDR (amino acids 209–679) and Axin analyses showed that the IDR II + III mutant can form small puncta (Fig. S4G) compared to the other truncated Axin mutants (data not shown). The purified recombinant APC–IDR–GFP fusion protein could assemble liquid droplets in vitro (Fig. S4H). A high concentration of PEG reduced the concentration required for droplet formation of APC–IDR recombinant protein in vitro (Fig. S4I). These findings indicated that APC also has a phase separation ability that enhanced the properties of the destruction complex condensates.

### Truncated APC mutants maintain the presence of the destruction complex condensates

**Fig. 2:**
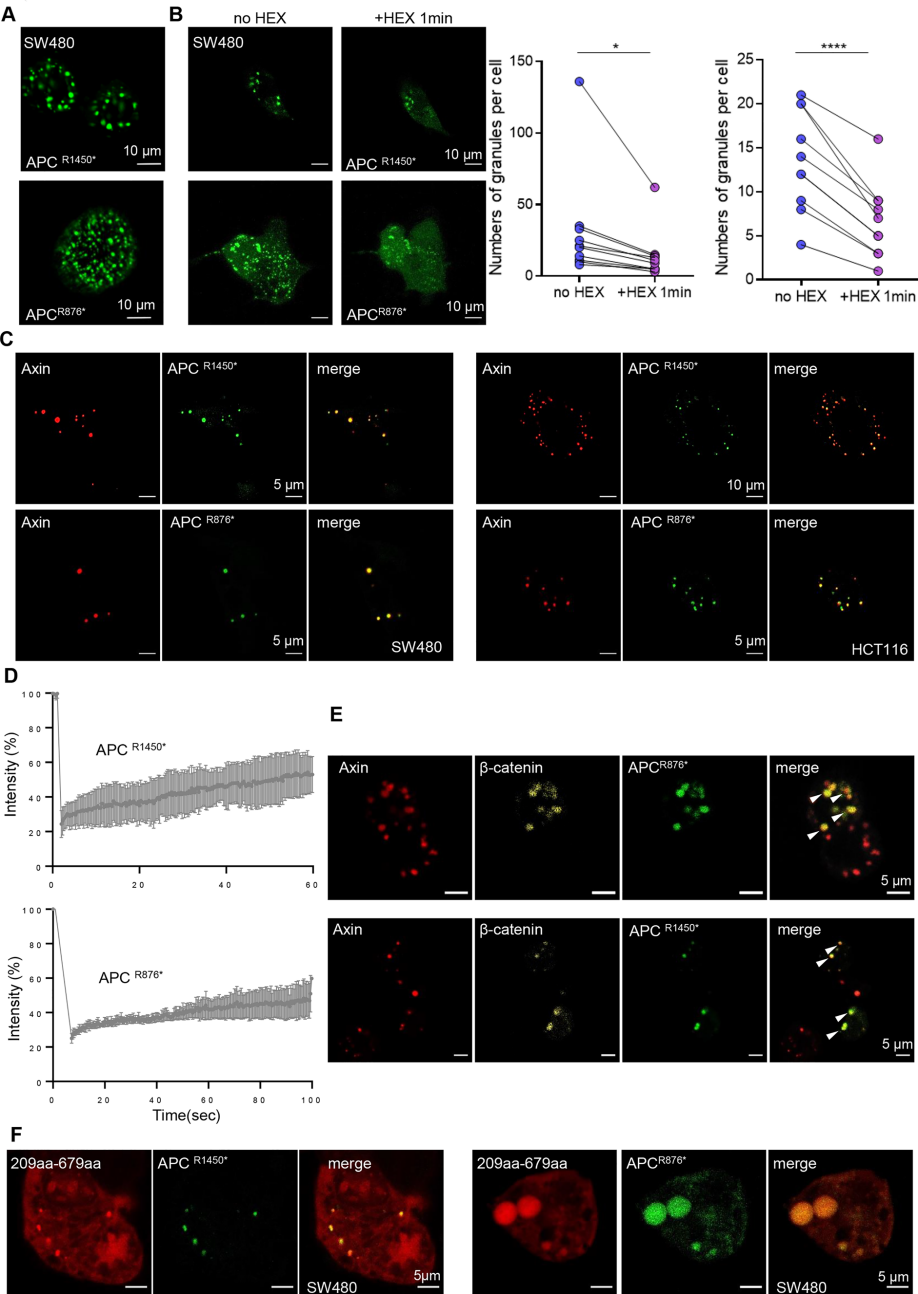
Truncated APC mutants maintain the presence of the destruction complex condensates. **A** Confocal images of intracellular truncation mutant EGFP-tagged APC protein in SW480. The truncated sites are aa1450 and aa876, respectively, named APC^R1450*^ and APC ^R876*^. **B** Left: representative images of the APC^R1450*^ and APC ^R876*^ cells treated with 5% 1,6-hexanediol (1,6-HD) for 1 min in CRC cells. Right: quantification of the number of APC^R1450*^ and APC ^R876*^ puncta per cell at the indicated times with 5% 1,6-HD. *n* = 8; *, *p* < 0.05; ***, *p* < 0.001. **C** Representative live-cell images of puncta formation transfected with Axin (red) and APC^R1450*^ (left, green) or APC ^R876*^ (right, green) in CRC cells. **D** FRAP analysis of Axin and truncated APC^R1450*^ (left) or APC ^R876*^ (right) puncta in CRC cells. Curves show the average time course of normalized fluorescence intensity. *n* = 5 cells. **E** Confocal images of live CRC cells expressing Axin (red), APC^R1450*^ (up, green), or APC ^R876*^ (down, green) and β-catenin (yellow) puncta. The arrow indicates representative colocalization puncta. **F** Representative images of live SW480 cells transfected with Axin-IDR (aa209-679) (red) and APC^R1450*^ (left, green) or APC ^R876*^ (right, green) proteins

As shown by cBioPortal (www.cbioportal.org) analysis, 12.7% (30/237) of the detected APC mutations were variants of uncertain significance (VUS), whereas 87.3% (207/237) were driver mutations. Among the driver mutations, 98.5% (204/207) were truncated mutations (Figs. S5A-B). The amino acid sites most prone to truncation mutations in APC were 1450 and 876 (Fig. S5A); hence, we constructed truncation mutant plasmids with an EGFP label named APC^R1450*^ and APC ^R876*^. Surprisingly, the mutant APC was still involved in the β-catenin destruction complex condensates in CRC cells (Fig. 2A). In addition, after 2.5% 1,6-hexanediol (1,6-HD) treatment, which disturbs weak intermolecular forces present in liquid-like assemblies [34], most granules disappeared (Fig. 2B). These results indicated that truncated APC does not impact the formation of destruction complex condensates. Interestingly, we found that most Axin granules persisted after 1,6-HD treatment (Fig. S3H). This finding further indicated that Axin, rather than APC, is the most important protein in the protein phase separation of the destruction complex.

Notably, we determined that Axin and the truncated APC proteins were absolutely colocalized into circular puncta (Fig. 2C and S5C). FRAP experiments indicated a rapid liquid-like recovery rate of the conjugated truncated APC proteins (Fig. 2D). Moreover, the truncated APC and Axin scaffold proteins could still recruit β-catenin to form biomolecular condensates (Fig. 2E), indicating that the destruction complexes were maintained even with truncated APC mutations in CRC cells. Then, we examined whether different domains of Axin could also colocalize with the truncated APC proteins. Our results showed that no puncta were observed except for Axin IDR (aa 209–679), whereas diffuse proteins that could not be fully recruited in the surrounding cytosol among granules were detected (Fig. 2F and Fig. S5D). Furthermore, we overexpressed the APC truncated mutants at the same time as Axin knockdown and found that although the APC truncated protein presented puncta, β-catenin was still diffusely distributed (Fig. S5E). In addition, in CRC cells with Axin knockdown, re-expressing wild-type Axin or Axin-IDR (aa 293–679) rescued condensate formation, whereas expressing Axin mutants that were defective in LLPS failed to rescue condensate formation (Fig. S5F). Together, these results indicated that the APC truncation mutation does not affect the formation of the β-catenin destruction complex condensates organized by Axin phase separation.

### Truncated APC mutations restrict Axin recruitment of GSK3β and CK1α into the destruction complex condensates

**Fig. 3:**
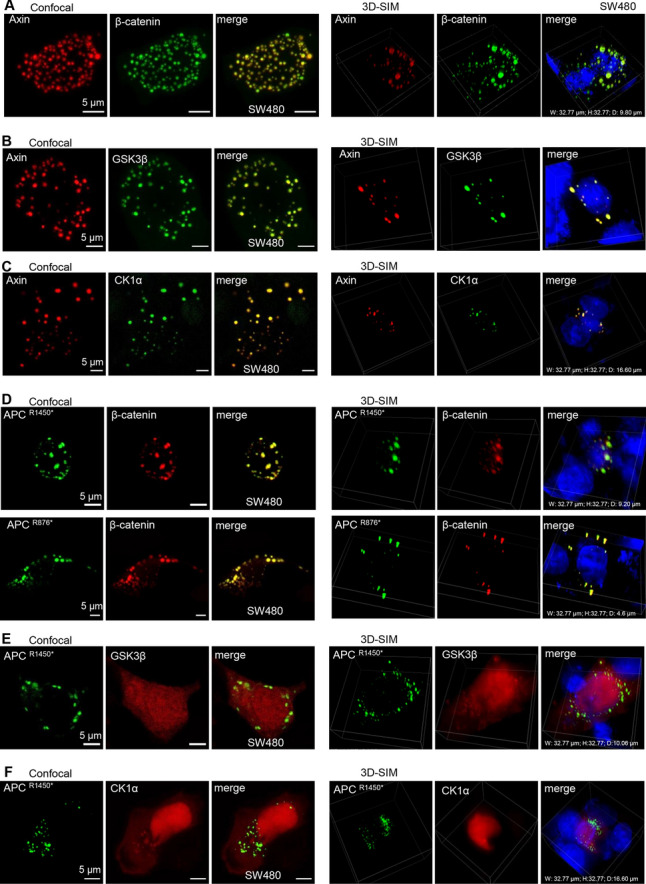
Truncated APC mutations restrict Axin recruitment of GSK3β and CK1α into the destruction complex condensates. **A**–**C** Left: the immunofluorescent images of live SW480 cells expressing Axin (red) and β-catenin (green) (**A**) or GSK3β (green) (**B**) or CK1α (green) (**C**). Right: 3D super-resolution structured illumination microscopy (SIM) spatially reveals homogeneous spheroid proteins. **D** Left: representative images of live SW480 cells expressing β-catenin* (red) and APC^R1450*^ (green) (up) or APC ^R876*^ (green) (down) puncta; right: representative 3D images spatially reveal homogeneous spheroid proteins. **E**–**F** Left: representative fluorescence images of live SW480 cells after transiently expressing EGFP-tagged APC^R1450*^ and mcherry-tagged GSK3β (**E**) or CK1α (**F**). Right: 3D-SIM spatially reveals different proteins

We further explored whether other client proteins colocalize in destruction complex condensates organized by Axin phase separation. As expected, the key Wnt-effector β-catenin and two kinases, GSK3β and CK1α, were both incorporated into the condensates in CRC cells (Fig. 3A–C and S6A), and truncated APC proteins could also bind β-catenin to form condensates (Figs. 2E and 3D). The condensates exhibited homogeneous spheroids, as revealed by 3D super-resolution structured illumination microscopy (SIM) (Fig. 3A–D and S6B–C).

**Fig. 4:**
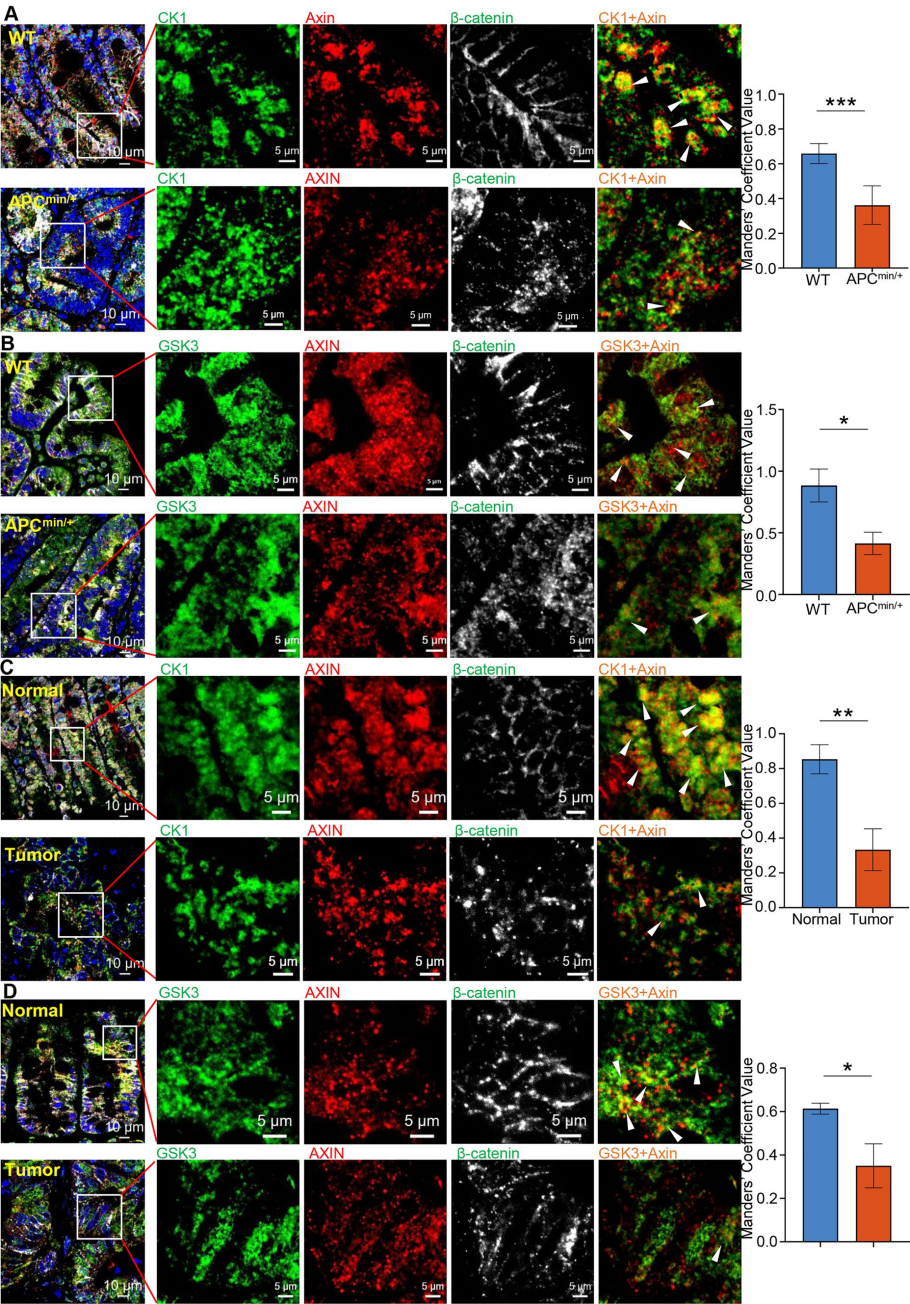
Multiplex immunofluorescence staining of CRC patient and APC^Min/+^ mouse adenoma tissues. **A**–**D** Left: multiplex immunofluorescence staining of Axin (red), β-catenin(white), CK1α (green), GSK3β (green), and nuclear (DAPI, blue) in the intestinal epithelial tissues of APC^Min/+^ mouse or wild-type mice (**A**–**B**), CRC patients and adjacent normal tissues (**C**–**D**). The arrows indicate representative the colocalization of Axin and CK1α or Axin and GSK3β regions. Right: quantitative determination of the correlation of colocalization between different proteins by Mander’s overlap coefficient (MOC) analysis in ten high power fields. Error bars indicate the SEM. *, *p* < 0.05; **, *p* < 0.001

Previous studies have suggested that disassembly of the destruction complex leads to β-catenin accumulation in the cytoplasm, and thus, transport into the nucleus abnormally activates the Wnt signaling pathway, and this process is often accompanied by truncated mutation of APC in CRC [35]. Surprisingly, GSK3β and CK1α were both unable to be incorporated into the condensates with APC truncated mutations, as shown by confocal microscopy and 3D-SIM (Fig. 3E–F and S6D–E), which may explain why APC mutation prevents the degradation of β-catenin. Moreover, truncated APC reduces the interaction between GSK3β and CK1α and destruction complex by Co-immunoprecipitation (Co-IP) (Figs. S6F-G). Indeed, multiplex immunofluorescence staining of CRC patient and APC^Min/+^ mouse adenoma tissues showed that Axin and GSK3β or Axin and CK1α exhibited fewer colocalized regions than those of the controls (Fig. 4). Taken together, these results revealed that truncated mutations in APC do not lead to disassociation of the destruction complex condensates but prevent two key kinases, GSK3β and CK1α, from being recruited, which helps elucidate the transport of β-catenin into the nucleus and the transcription of Wnt-responsive genes in CRC.

### Axin is involved in the β-catenin nuclear translocation and transcriptional function as a droplet

**Fig. 5:**
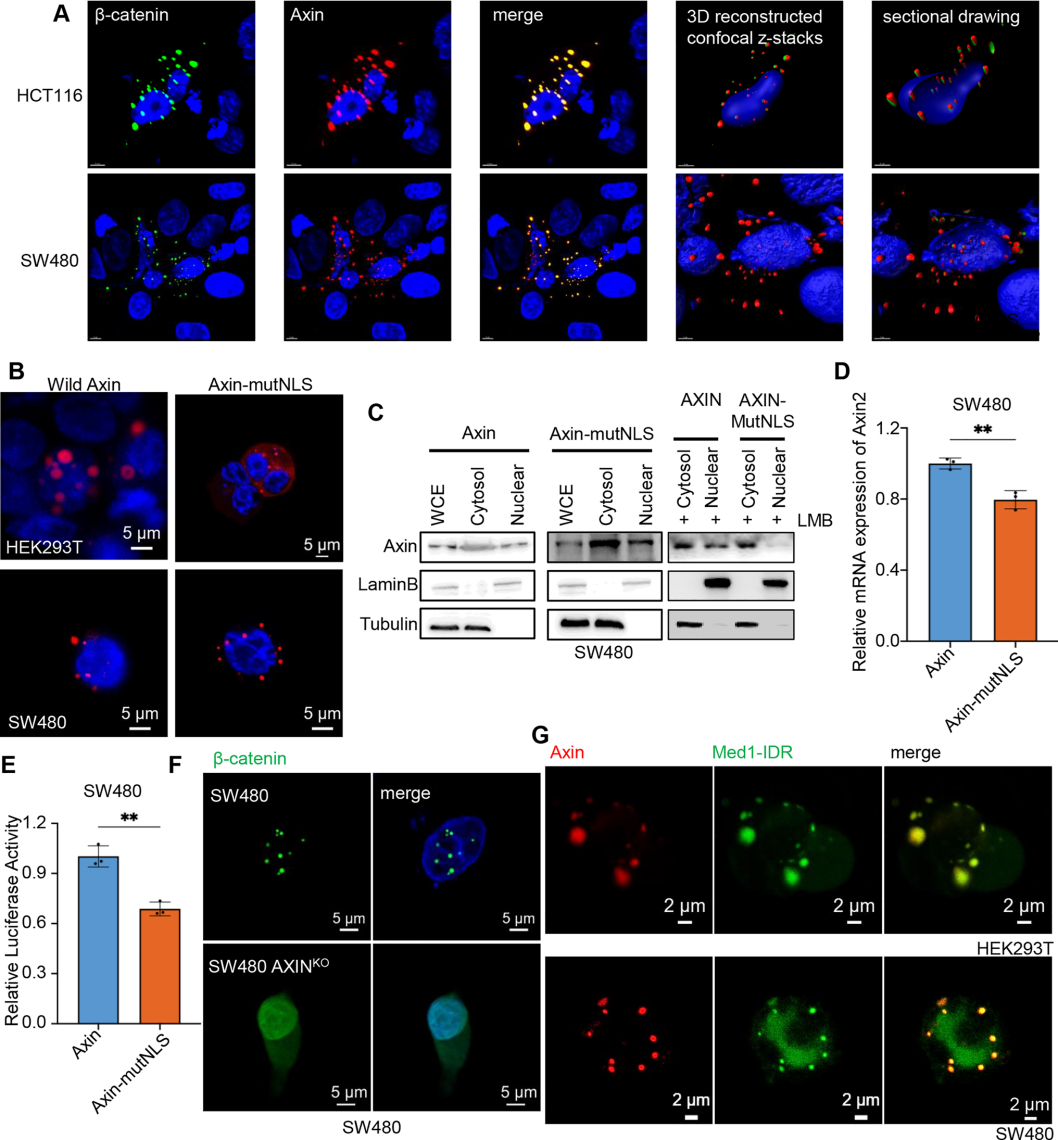
Axin is involved in the β-catenin nuclear translocation and transcriptional function as a droplet. **A** Confocal images of intracellular Axin (red) and β-catenin (green) in CRC cells. Line four: representative images of 3D reconstitution using by Imaris software. Line five: section drawing of 3D reconstructed confocal images by Imaris software. The tool, Clipping Plane, which allows to re-slice in any chosen rotation (https://imaris.oxinst.com/), uncovers hidden regions by re-slicing the image data in a different orientation. **B** Representative images of Axin (red) and nuclear (DAPI, blue) expression after transfection of NLS-mutated Axin in HEK293T and SW480. **C** Cells were treated with or without Leptomycin B (LMB, 200 nM, 24 h). Nuclear and cytoplasmic protein extraction experiment was further performed to measure the cellular localization of Axin in SW480 cells after transfection of NLS-mutated Axin. **D** The mRNA levels of Wnt pathway-specific downstream target gene (Axin2) were examined by RT-PCR in SW480 cells expressing wild-type Axin or NLS-mutated Axin. Error bars, mean ± SD; **, *p* < 0.01. **E** Analysis of TOP/FOP reporter activity in SW480 cells transfected with wild-type Axin or NLS-mutated Axin. Error bars, mean ± SD; **, *p* < 0.01. **F** Representative images of β-catenin (green) and nuclear (DAPI, blue) expression after transfection of β-catenin in Axin KO SW480 or wild-type SW480 cells. **G** Representative fluorescence images of live SW480 and HEK293T after transiently expressing EGFP-tagged Med1-IDR and pScarlet-tagged Axin

Notably, we found that the images using Imaris software with 3D reconstructed confocal z-stacks showed that Axin and β-catenin are colocalized to the nucleus in a condensation manner in CRC cells (Fig. 5A). To determine whether β-catenin is imported by an NLS-dependent mechanism rather than an Axin-dependent mechanism, we used PSORT II (https://psort.hgc.jp/), an independent subcellular localization predicting program to analyze the β-catenin to predict that the amino acid sequences at positions the 667aa to 673aa (^667^PQDYKKR^673^) were NLS (Fig. S7A). And the NetNES web server used to predict the nuclear export signals showed that the NES were at positions 8aa to 14aa (^8^MELDMAM^14^) (Fig. S7B). Then, we linked an additional standard NLS sequence or overexpressed the mutant NLS/NES sequence (Fig. S7C), and β-catenin did not change its localization in the cell and remained in puncta in the nucleus independent of the classic NLS or NES (Fig. S7D), consistent with the previous studies [22, 25]. β-Catenin enters the nucleus through a non-classical pathway, and Axin may assist β-catenin into the nucleus.

Indeed, two NLS of Axin were predicted by PSORT II program (Fig. S8A), and then, the truncated mutations containing two NLS sequences were constructed, respectively (Fig. S8B). Truncated Axin containing the first NLS (^344^PPYRIRK^351^) could enter the nucleus; however, the other protein is mainly expressed in the cytoplasm (Fig. S8C). When we overexpressed the mutant NLS, the Axin had higher expression content in the cytoplasm compared with wild-type Axin (Fig. 5B), and this observation was also supported by the results of cell fractionation experiments (Fig. 5C). We also found that Leptomycin B (LMB) treatment did not affect the subcellular localization of Axin, suggesting that lower expression of Axin in nuclear did not affect the nuclear export (Fig. 5C). These results suggest that the amino acid sequences at positions the 344aa to 351aa (^344^PPYRIRK^351^) were the functional localization signal sequence of Axin. Further, we investigated whether mutant NLS in Axin affects the transcription of Wnt-responsive genes. The expression of Axin2, which is often used as an indicator of canonical Wnt pathway activity [36], was examined. QPCR and TOP/FOP-Flash reporter assay showed overexpression of Axin-mutNLS inhibited Wnt/β-catenin signaling activation (Fig. 5D–E and S8D–E). To investigate whether the accumulation of β-catenin is critically related to Axin in the nucleus, we reduced the expression of Axin in cells. The results showed that the knockout or knockdown of Axin induced unsuccessful β-catenin granule formation in cells (Fig. 5F and S8F). These findings suggested that Axin plays an important role in the formation of β-catenin aggregates in the nucleus.

It has been reported that transcription factors can interact with Mediator through the phase-separating capacity and form the condensates with Mediator to participate in gene activation [37]. And β-catenin was incorporated and concentrated into transcriptional condensates formed at super-enhancers [26]. We detected whether Axin would also be incorporated into the super-enhancers. The results showed that Axin colocalized with Med1-IDR which could form nuclear puncta at super-enhancers[38], suggesting that Axin could influence transcription by co-integrating with β-catenin into super-enhancers, even in cells with APC mutations (Fig. 5G). These data together suggest that Axin may facilitate the progress of recruitment and agglutination of β-catenin into transcriptional condensates.

## Discussion

Evidence increasingly shows that LLPS is the basis for membraneless cells compartments, expanding our understanding of cell biology processes [39, 40]. The Wnt signaling pathway plays an important role in the regulation of cell fate specification and proliferation [41]. Hyperactive Wnt signaling can result in malignant tumors, and the cytoplasmic β-catenin destruction machinery is tightly controlled to prevent this process [42]. However, the mechanisms that underlie the dynamic formation and dissolution of the destruction complex remain undefined. Surprisingly, a recent study and our data have shown that the β-catenin destruction complex exhibits properties of liquid droplets. Scaffold molecules that drive phase separation are mostly characterized by IDRs with multiple interacting motifs [43–45]. Our results demonstrated the IDRs (209aa-679aa), which contains binding sites with β-catenin and GSK3β, was the critical region to drive Axin phase separation in CRC cells. This surprisingly long disorder region could have huge and multiple influences on the function and structure of Axin. Consequently, another challenge is to more fully explore which parts of the IDRs of Axin are critical for their functions, which act to improve efficiency, and which may be redundant in CRC cells, and this applies equally to the APC IDR. Axin and APC as scaffold proteins undergo LLPS to form destruction complex condensates which recruit β-catenin, CK1a, and GSK3β to the droplets, where β-catenin is degraded. APC could stabilize the destruction complex and increase the dynamics of Axin condensates [3, 6, 33]. We also identified new domains of Axin and APC that drive phase separation, providing more detailed insights into the molecular mechanisms of malignant tumors associated with Wnt pathway activation.

Mutations inactivating the APC gene are found in approximately 80% of all human CRCs. However, it is unclear how APC mutations impact the β-catenin destruction complex condensates. LLPS may recruit molecules to increase reaction rates. If one critical component is recruited into a dense phase but all other components for signaling events remain in the dilute phase, the signaling event will be inhibited or slowed [46]. In this study, we determined that despite the absence of all the SAMP motifs that contain Axin-binding sites [47], the truncated APC and Axin still colocalized and formed liquid droplets. This result indicated other unknown interacting sequences between the Axin and APC scaffolds. In addition, truncated APC could still bind β-catenin but could not promote its phosphorylation, ubiquitination, and eventual proteasomal degradation. Research has shown that truncated APC still phosphorylates β-catenin [48], but our results demonstrated that neither GSK3β nor CK1α, two key kinases, can be recruited into liquid droplets in the APC mutant CRC cells. Thus, membraneless compartments separate β-catenin from these kinases, resulting in the accumulation of β-catenin in the cytoplasm and translocation into the nucleus, where it binds to TCF/LEF [49] to form the β-catenin/TCF transcription complex [50].

The nuclear localization of β-catenin is the defining step in Wnt pathway activation. β-Catenin has no identifiable NLS^23^ and enters the nucleus via a non-classical pathway [51]. Nonetheless, the mechanisms regulating β-catenin nuclear transport remain undefined. Interestingly, we found that β-catenin and Axin colocalize with droplet properties in the nucleus. In addition, when Axin was genetic knockdown or CRISPR knockout, β-catenin was distributed diffusely in both cytoplasm and nucleus. The evidence described here argues that β-catenin trafficking into the nucleus may be associated with liquid-like properties expression of Axin in the nucleus. Moreover, phase separation condensates formed by transcription factors and the Mediator coactivator is involved in the transcription apparatus at super-enhancers [10, 12, 37, 52], including β-catenin incorporated into the condensates [26]. Our results suggest another possibility that droplet properties of Axin in the nucleus may play an important role in the recruitment of β-catenin into the transcriptional condensates, which deserves considerable effort to confirm. Targeting oncogenic Wnt signaling pathways in which Axin and β-catenin complex may generally interact with super-enhancer condensates could present a therapeutic opportunity for CRCs.

**Fig. 6:**
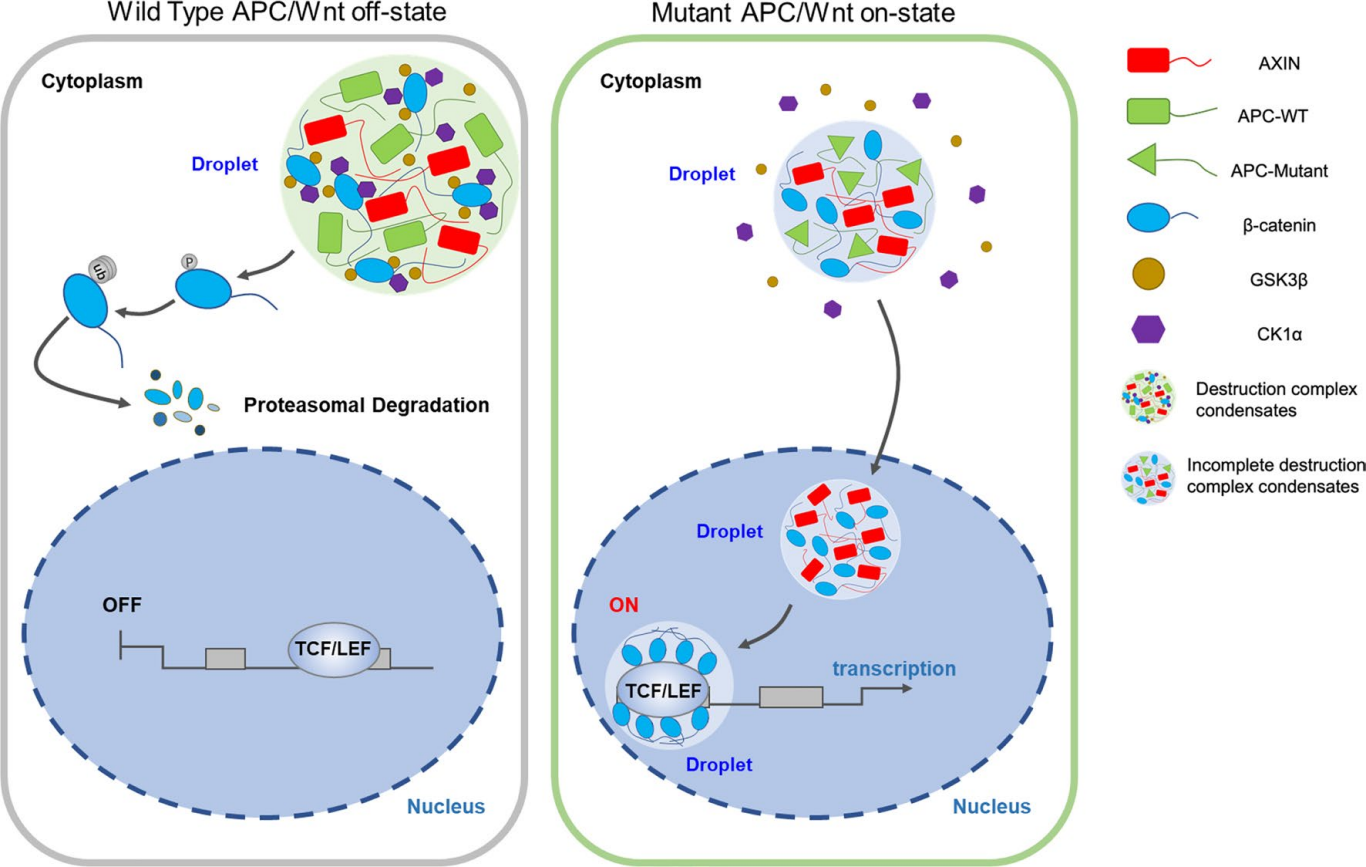
Schematic representation of this study. APC mutations disrupt the β-catenin destruction complex condensates organized by phase separation in CRC cells. And truncated APC did not affect the formation of condensates, but GSK 3β and CK1α were unsuccessfully recruited and resulting in accumulation in the cytoplasm of CRCs. Furthermore, Axin participated in the β-catenin nuclear translocation and recruitment into the transcription condensation, driving the progression of CRC

In conclusion, our work reveals a novel mechanism by which APC mutations disrupt the β-catenin destruction complex condensates organized by phase separation in CRC. We demonstrated that the formation of destruction complex condensates depends on the β-catenin accumulation of high concentrations, APC mutations prevent Axin from recruiting GSK3β and CK1α into the condensates, and Axin is related to the β-catenin nuclear translocation and its transcriptional function as a droplet, which could drive CRC initiation and progression (Fig. 6).

### Supplementary Information

Below is the link to the electronic supplementary material.

Supplementary file1 (PDF 1490 KB)

Supplementary file2 (MP4 2663 KB)

## Data Availability

All the other data supporting the findings of this study are available within the article and its Expanded Information files and from the corresponding author upon reasonable request.
