# Supplementary material for: APC mutations disrupt β-catenin destruction complex condensates organized by Axin phase separation
Source: Cell Mol Life Sci. 2024 Jan 27;81(1):57. doi: 10.1007/s00018-023-05068-0 (PMC10817841; doi:10.1007/s00018-023-05068-0)
Supplement: Supplementary file 1 — Supplementary file1 (PDF 1490 KB) [file 18_2023_5068_MOESM1_ESM.pdf]

Supplementary materials and methods

Plasmids, antibodies and reagents

pScarlet-N1-Axin (N/A) was purchased from Mail Gene (Beijing); pReceiver-M01 APC ^{R876*} (CS-Y3960-M01-01), pReceiver-M01 APC ^{R1450*} (CS-Y3960-M01-02), pReceiver-M01 APC SAMP-1(CS-Y3960-M01-03), pReceiver-M01 APC SAMP-2 (CS-Y3960-M01-04) , pReceiver-M01 APC-IDR(CS-Y3960-M01-05), pScarlet-N1 RGS(CS-E2160-pScarlet-01), pScarlet-N1 GSK3B binding site(CS-E2160-pScarlet-02), pScarlet-N1 CTNNB1 binding site(CS-E2160-pScarlet-03), pScarlet-N1 DIX(CS-E2160-pScarlet-04), pScarlet-N1 TNK(CS-E2160-pScarlet-05), pScarlet-N1 predicted IDR II +III (CS-E2160-pScarlet-06), pScarlet-N1 predicted IDR II (CS-E2160-pScarlet-07), pScarlet-N1 predicted IDR III (CS-E2160-pScarlet-08), pScarlet-N1 predicted IDR I(CS-E2160-pScarlet-09), pScarlet-N1 Axin IDR(CS-E2160-pScarlet-10), pReceiver-B31 Axin(EX-E2160-B31), pEZ-M56 CTNNB1-mcherry(EX-T0573-M56), pReceiver-M33 GSK3B -CFP(EX-Z0294-M33), pReceiver-M33 CTNNB1-YFP(EX-T0573-M16) and pScarlet-N1 CSNK1A1(CS-Z6003-pScarlet) were purchased from Gene Copoeia (Shanghai); pET-21a (+)-APC IDR -Linker GFP-8HIS(N/A), pET-21a (+)-Axin IDR-Linker mScarlet-GST(N/A), pLenti-U6- spgRNA v2.0-CMV-Puro-P2A-3Flag-spCas9-Axin(N/A), pLenti-U6-spgRNA1 v2.0- U6- spgRNA2 v2.0-CMV-Puro-P2A-3Flag-spCas9-APC (N/A), pAdeno-Axin donor (N/A) and pAdeno-APC donor (N/A) were purchased from OBIO Technology(Shanghai). Axin-ΔIDR (Δ209-530aa), Axin-ΔIDR (Δ531-679aa), Axin-ΔIDR (Δ209-679aa), Axin-ΔID (Δ209-464aa + Δ497-679aa), Axin-ΔIDR (Δ465-496aa), Axin-513, Axin-862, Axin NLS mut, β-catenin NLS mut, Med1-IDR are constructed by our laboratory. The following antibodies were used at 1:1,000 for Immunofluorescence: rabbit anti-Beta Catenin (Proteintech; Cat#66379-1-Ig; RRID: AB_2857358), rabbit anti-GSK3B (Proteintech; Cat#22104-1-AP; RRID: AB_2878997), rabbit anti-Axin((Proteintech; Cat#16541-1-AP; RRID: AB_2062409) and rabbit anti-Casein Kinase 1 alpha (Affinity Biosciences; Cat#DF3185; RRID: AB_2835408). Opal™ 7-Color Manual IHC Kit was from Akoya Bio (NEL811001KT);

Cell culture and transfection

HCT116, HCT15, HEK293T, RKO, and SW480 cells were obtained from the American Type Culture Collection and tested negative for mycoplasma contamination. HCT116, HCT15, and HEK293T cells were cultured in DMEM (Gibco, C11995500BT) with 10% FBS (Vistech, SE100-011), 100 units/mL

penicillin and 100 mg/mL streptomycin; the other cells were grown in RPMI 1640(Gibco, 11875176) with 10% FBS, 100 units/mL penicillin and 100 mg/mL streptomycin. All cells were cultured at 37°C in a humidified atmosphere of 5% CO_2. Transient transfections were performed with Lipofectamine 2000 (Invitrogen, 11668019) following the manufacturer's guidelines. When double transfection was needed, equal amounts of both plasmids were simultaneously transfected.

CRISPR/Cas9 knock-in in cells

For introduction of pScarlet at the N-terminus of Axin CRISPR/Cas9 lentivirus and Axin donor lentivirus were purchased from Obio Technology (Shanghai, China). Axin guide RNAs (gRNAs) were designed using the online CRISPR design tool (http:// CRISPR.mit.edu/). The Axin gRNA sequences was GTGAGCGACGAGTTTGACTGTGG. The plasmids were 3FLAG pLenti-U6-spgRNA v2.0-CMV-Puro-P2A-3Flag-spCas9 and pAdenoMCMV-MCS. The experiment was performed according to the lentivirus manual. Cells were selected and plated as single cells in a 96-well plate using a BD Fortessa system. After several weeks of incubation, wells containing single-cell colonies were used to expand the culture, and DNA was extracted for genotyping using the Animal Tissue DNA Isolation Kit (Fore Gene). For determination of the copy number of correct gene edits, the genomic sequence of the genes of interest was sequenced by Ruibiotech.

Production of stable Axin or CTNNB1 KO cell lines

Axin and CTNNB1 guide RNAs (gRNAs) were designed using the online CRISPR design tool (http:// CRISPR.mit.edu/). The Axin gRNA sequence was gRNA AAACTTGCTCCGAGGTCCAA. The CTNNB1 gRNA sequence was gRNA-a CTGGCAGCAACAGTCTTACC. The gRNAs were cloned into the pCRISPR-LvSG06. CRISPR/Cas9 lentivirus were purchased from GeneCopoeia (Guangzhou, China). Lentivirus from HEK293T cells containing the CRISPR-Cas9 system was used to infect cells. To obtain single clones of Axin、CTNNB1 KO cells, cells selected with puromycin for 3 days and isolated by colony formation assay or single cell culture. The single clones were validated by immunoblotting analysis and DNA sequencing.

TOP/FOP-Flash reporter assay

The TOP/FOP-Flash was co-transfected into cells along with wild-type Axin or NLS-mutated Axin in Axin KO SW480 cells. After transfection for 48 h, the cells were harvested for analysis with the Dual-Luciferase Reporter Assay System (Promega, USA) according to the manufacturer's instructions. The

luciferase activity was measured using the PerkinElmer EnSpire Multilabel Reader 2300 (PerkinElmer, USA). The luciferase intensity was normalized to the Renilla luciferase activity to standardize for transfection efficiency.

RNA isolation and real-time quantitative PCR

The total RNA was performed using SevenFast total RNA extraction Kit (SM132, SEVEN Biotech) following the manufacturer's protocols and reverse-transcribed into cDNA using One-Step gDNA Removal (AU341-02, Transgen). Real-time PCR was performed using SYBR qPCR Master Mix (Q711, Vazyme) with an Applied Biosystem 7500 instrument (Applied Biosystems). Relative expression to GAPDH was determined using the $2^{-\Delta\Delta Ct}$ method. The primers used are below. Axin2:

Sense 5'-3(CAAACTTTCGCCAACCGTGGTTG);

Antisense 5'-3': (GGTGCAAAGACATAGCCAGAACC).

Western blot and Co-immunoprecipitation (Co-IP)

Total protein was extracted using radioimmunoprecipitation assay buffer (50 mmol/L Tris-HCl pH 8.0, 150 mmol/L NaCl, 0.5% Na-deoxycholate, 0.1% sodium dodecyl sulfate, 1mM PMSF). Protein concentration was determined by BCA Protein Assay Kit (KGP902, KeyGEN). Equal amounts of protein lysates were separated by SDS-PAGE gel and electrotransferred to PVDF membrane. After blocking in 5% milk-TBST for 1 hour at room temperature (RT), the membrane was incubated with primary antibodies, including anti α-tubulin (Rayantibody), anti-LaminB (Proteintech), anti-Axin (Proteintech) at 4 °C overnight and secondary antibodies at RT for 45min. Images were acquired using a ChemiDoc™ Imaging System (Bio-Rad, USA).

For Co-IP, SW480 cells were cultured in a 100 mm culture dish. Axin-Flag, APC ^{R876*}-GFP and APC ^{R1450*}-GFP were transfected respectively. 1× lysis buffer supplemented with PMSF. After centrifuging at 12,000×g for 15 min at 4 °C, per 300 μL of the supernatant was added with 20 μL Protein A/G PLUS-Agarose (Santa Cruz) and incubated with the Anti-Flag antibody (Dia-an) or anti-GFP antibody (ImmunoWay) overnight at 4 °C. The next day, the immunoprecipitants were washed five times at 2000×r for 2 min at 4 °C and boiled with 1× Loading Buffer. At last, Western blotting assay was conducted according to the protocol above. Here, IgG (Proteintech) was used as a negative control.

Cell fractionation

4×10^7 SW480 cells were harvested and lysed by Triton X-100. Nuclei were collected in pellet 1 by

low-speed centrifugation (5 min, 12,000rpm, 4°C). The supernatant was further clarified by high-speed centrifugation (10 min, 12,000rpm, 4°C) to remove cell debris and insoluble aggregates. Nuclei were washed once in buffer A and then lysed. The resulting two fractions were re-suspended in the Laemmli buffer, boiled for 15min and resolved by SDS-PAGE for western analysis.

Immunofluorescence (IF) and confocal microscopy

Cells were seeded in a 24-well plate (Corning) with a coverslip in each well. The following day, the cells were fixed for 30 minutes with 4% PFA and treated with 0.5% Triton X-100 for 20 minutes at room temperature. After normal goat serum (ZSGB-Bio) blocking for 30 minutes at 37°C, the cells were incubated with primary antibody for 16 h at 4°C. After PBS washes, the corresponding fluorochrome-labeled secondary antibody was applied for 30 minutes at room temperature in the dark, and the samples were washed and then mounted with DAPI (Sigma, D9542). Following washes in PBS, coverslips were mounted onto slides with Vectashield (Solarbio) and sealed using transparent nail polish. Images were acquired on a confocal laser-scanning microscope (Zeiss LSM880, 63× oil objective, NA 1.4, 1 Airy Unit).

For multiplexed IF, it was carried out by using the PerkinElmer-Opal-Kit (Akoya Biosciences) according to the manufacturer's instructions. Briefly, these FFPE tissues were dewaxed with xylene, then rehydrated through a graded ethanol series, and fixed with neutral-buffered formalin prior to antigen retrieval that was performed with AR6 Buffer using high-pressure incubation. This step was followed by cooling, blocking, and serial staining with primary antibodies, HRP-conjugated polymers, and opal fluorophores; cycles were repeated until all markers were stained. Finally, the nuclei were counterstained with DAPI. Images were acquired on a confocal laser-scanning microscope (Zeiss LSM880).

Video 1 legend

Axin overexpression in SW480 cells induces cytoplasmic liquid condensates, related to Supporting Figure 3F.

Supplementary Figures and Figure legends

Fig S1

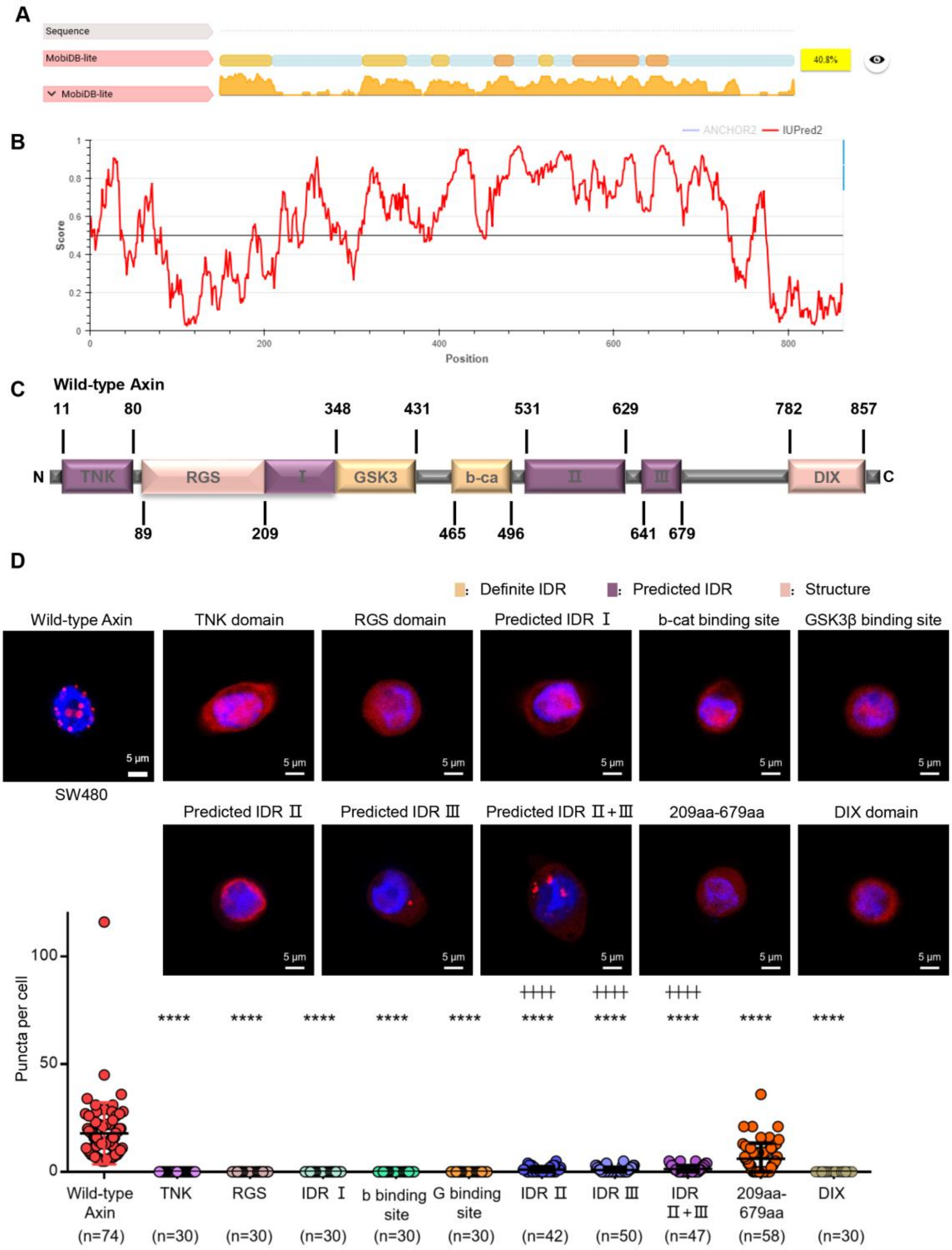

Figure S1

(A-B) Predictions and annotation for intrinsically disordered Axin protein by the MobiDB database (A) and IUPred web (B). The result indicate that Axin is a complex multidomain scaffolding protein containing large IDRs.

(C) Schematic diagram of domain structure of wild-type Axin 's full-length according to MobiDB database. Purple represents the predicted intrinsically disordered region; Yellow represents definite IDR; Pink represents structured residues.

(D) Comparison of Axin puncta numbers in the CRC cells expressing pScarlet-tagged wild-type or mutant Axin proteins. We generated a panel of pScarlet-tagged mutants containing only a single domain or predicted disordered region and assessed their ability to form puncta when overexpressed in CRC cells. No puncta were observed with the mutants lacking the IDR, in contrast to the wild-type control. Furthermore, the predicted IDR I, β-catenin binding site and GSK3β binding site alone did not form a droplet. Fluorescent puncta were detected in CRC cells expressing a truncation mutant containing the whole IDR (aa 209–679). Horizontal lines indicate the mean ± SEM. Representative fluorescence images of the SW480 cells transiently transfected with the pScarlet-tagged Axin constructs are shown above each bar. †††† $p < 0.0001$ vs IDR (aa209-679), ****$p < 0.0001$ vs wild-type Axin.

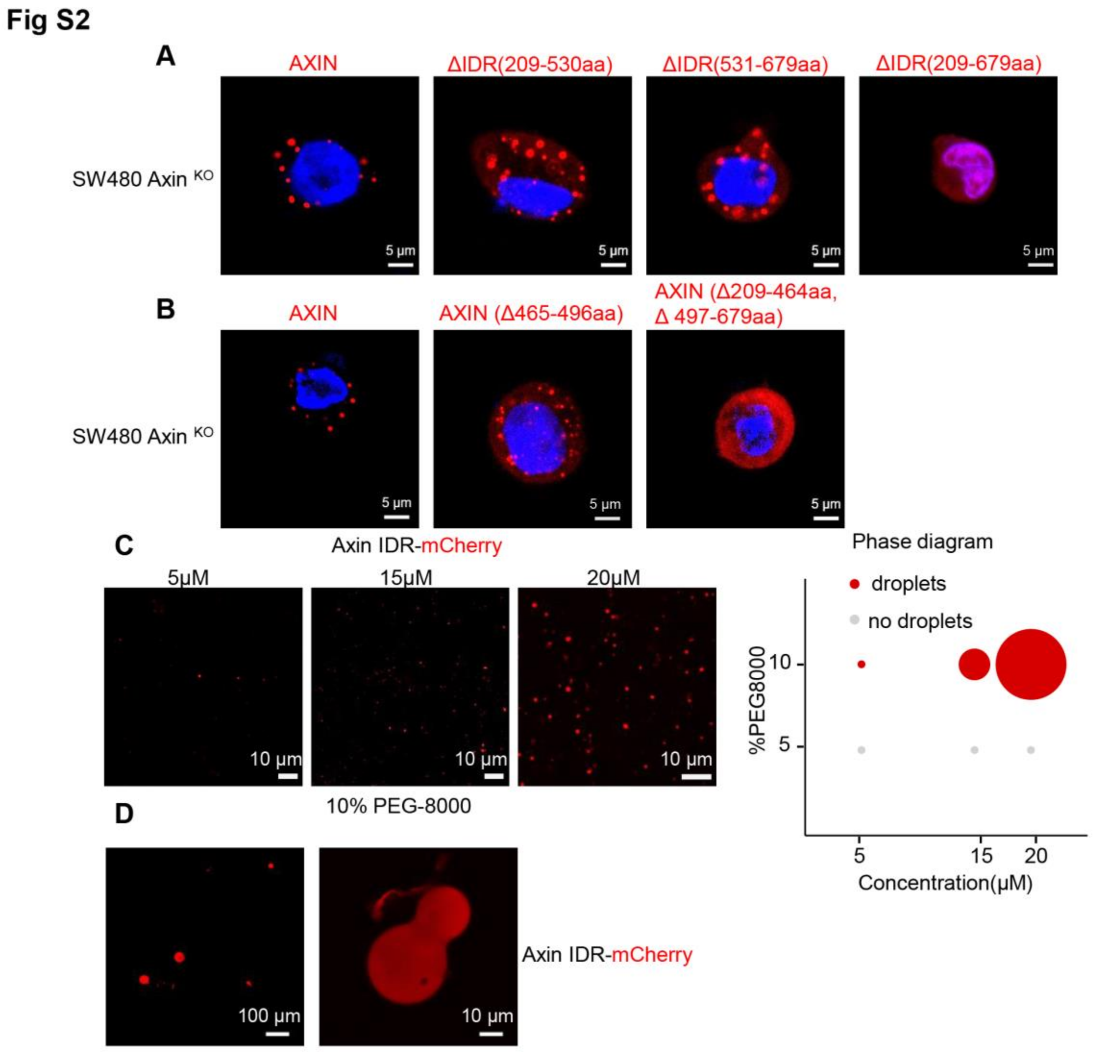

Figure S2

(A) Representative images of pScarlet -tagged mutants lacking the IDR in Axin KO SW480 cells. Mutants lacking the whole IDR (209–679 aa) failed to form puncta, while the lacking the 209–530 aa and 531-679 aa IDR still formed puncta in cells, indicating that IDR (209–679 aa) is critical for puncta formation, which is inconsistent with the reported article[1].

(B) Confocal images of pScarlet -tagged mutants lacking the IDR in SW480. Mutants lacking the whole IDR (209–679 aa) or part of IDR (aa 209–464 and aa 497-679) failed to form puncta, while the lacking the IDR-1 (aa465-496) which is the interaction region (aa 465-496) between Axin and β-catenin still formed puncta in cells.

(C) Left: representative fluorescence images of puncta formation by purified Axin-IDR (aa 209–679)-mCherry at the indicated concentrations. Right: phase diagram of Axin-IDR (aa 209–679)-mCherry in

the presence of various concentrations of PEG-8000. The size of the circle is proportional to the size of puncta detected under the respective buffer conditions.

(D) Representative images of the purified Axin-IDR mCherry fusion protein. Results C and D indicated that the Axin IDR (aa 209–679) can independently form condensates in vitro.

Fig S3

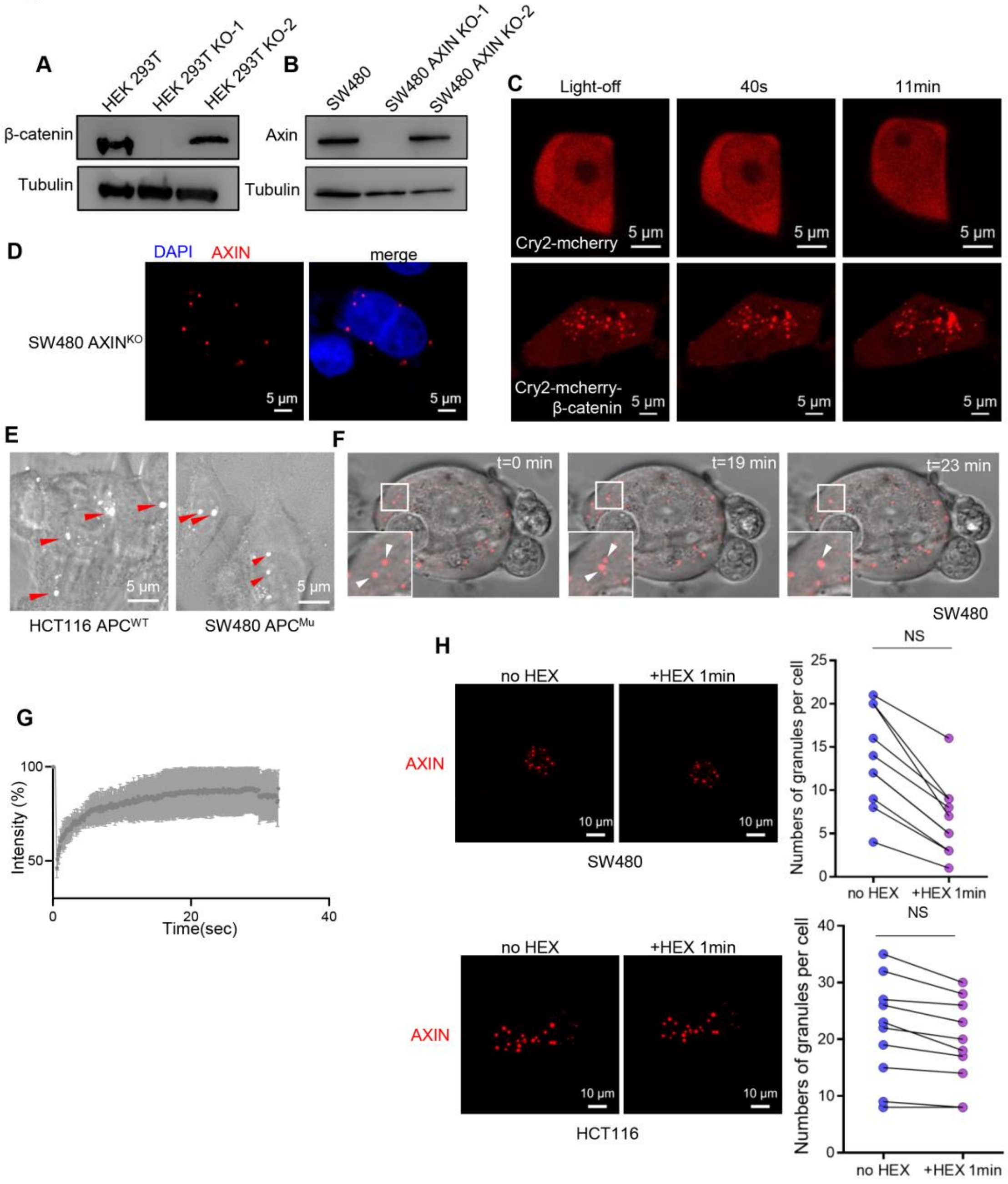

Figure S3

(A-B) The CRISPR/Cas9 system was applied to generate CTNNB1 KO cell from HEK293T (A) cells and Axin KO cell lines from SW480 cells (B). The efficiency of CTNNB1 KO and Axin KO were examined by Western-blotting. CTNNB1 KO-1 and Axin KO-1 are the two KO clones used in this study.

(C) Representative images during OptoDroplet activation in β-catenin ^{KO} HEK293T cells expressing Cry2-mcherry-β-catenin and Cry2-mcherry. Time intervals between illuminating blue light and image

acquisition are noted on each image.

(D) Representative images of Axin puncta formation after transiently expressing Axin in $Axin^{KO}$ SW480 cells.

(E) Observation of the localization of endogenously expressed Axin in CRC cells with or without the APC mutation using CRISPR–Cas9 knock-in. The arrow indicates representative Axin puncta.

(F) Time-lapse images of Axin puncta showing a droplet fusion event in SW480 cells after transiently expressing Axin-pScarlet. Cells were stimulated with a 561 nm laser every 10 s for 2 h.

(G) FRAP analysis of Axin puncta in SW480 cells. Curves show the average time course of normalized fluorescence intensity. n=5. Data is displayed as the mean ± SEM.

(H) Representative images of SW480-Axin cells and HCT116 cells with 5% 1,6-hexanediol (hex) treatment for 1 min.

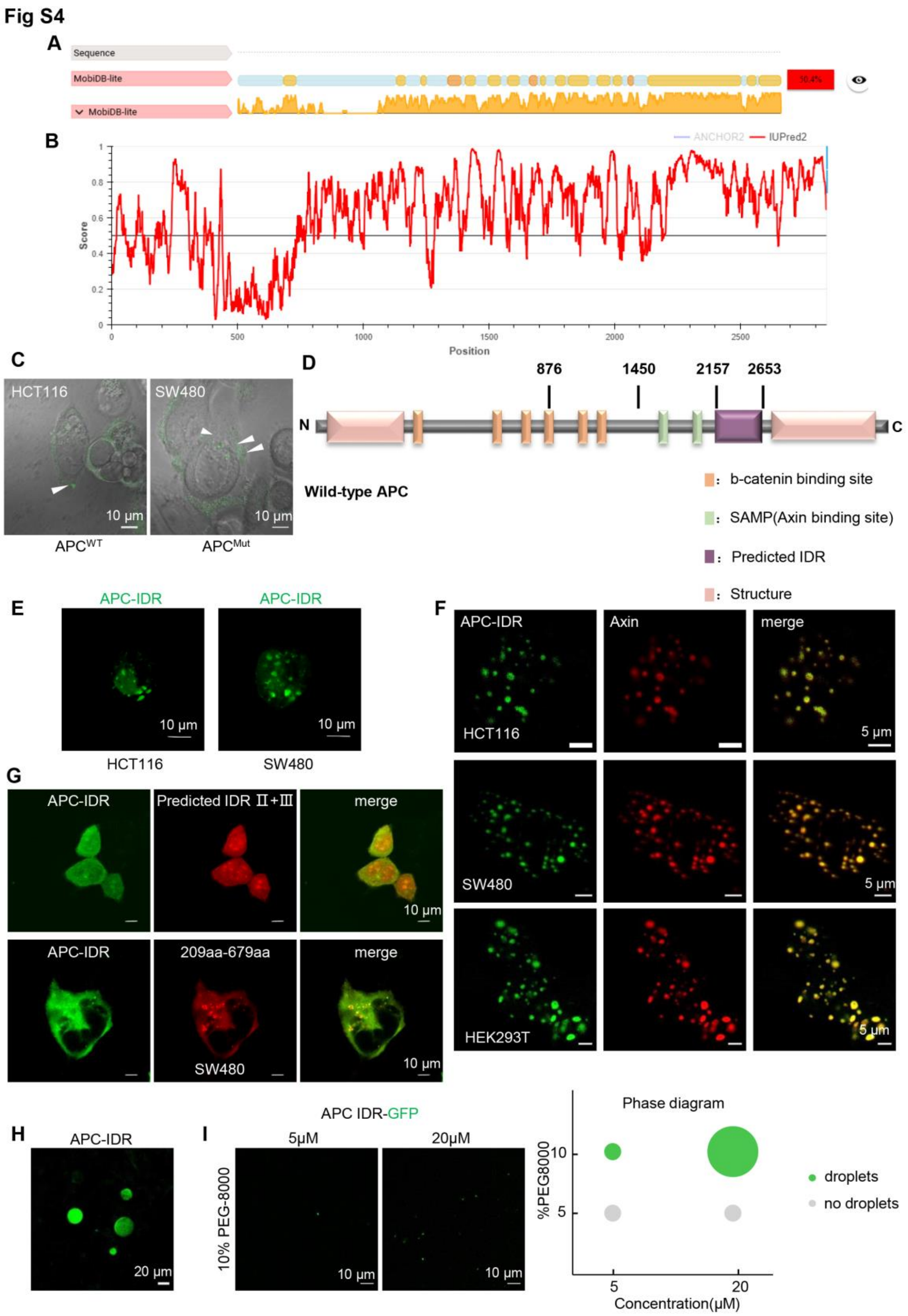

Figure S4

(A-B) Predictions and annotation for intrinsically disordered APC protein by the MobiDB database (A) and IUPred web (B). The result indicates that APC is also a complex multidomain scaffolding protein

containing large IDRs.

(C) Observation of the localization of endogenously expressed APC in CRC cells with or without the APC mutation using CRISPR–Cas9 knock-in. The arrow indicates representative APC protein puncta.

(D) Schematic diagram of domain structure of wild-type APC's full-length according to MobiDB database. Yellow represents b-catenin binding site; Green represents Axin binding site; Purple represents the predicted intrinsically disordered region; Pink represents structured residues.

(E) Representative images of the CRC cells expressing the APC-IDR protein (green).

(F) Representative fluorescence images of live CRC cells and HEK293T cells transfected with EGFP-tagged APC-IDR and pScarlet-tagged Axin.

(G) Representative fluorescence images of live SW480 transfected with EGFP-tagged APC-IDR and pScarlet-tagged mutant Axin.

(H) Liquid droplets were observed by wide-field fluorescence microscopy in purified APC-IDR protein (labeled with EGFP, green) solution in vitro.

(I) Left: Representative fluorescence images of puncta formation by purified APC-IDR-EGFP at the indicated concentrations. Right: phase diagram of APC-IDR-EGFP in the presence of various concentrations of PEG-8000. The size of the circle is proportional to the size of the puncta detected under the appropriate buffer conditions.

Fig S5

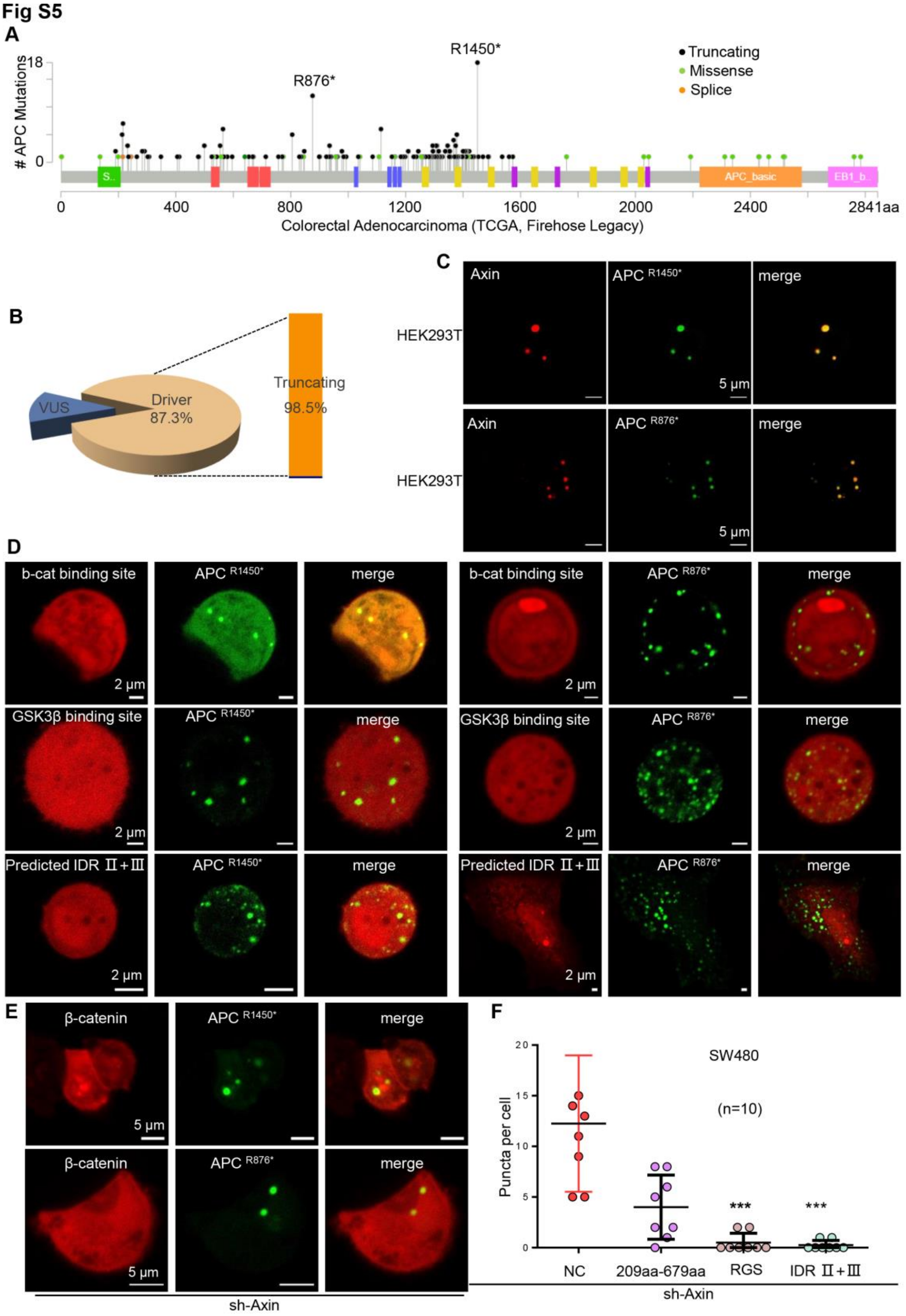

Figure S5

(A)The graphical view shows the APC protein domains and the positions and species of specific mutations. The length of the line connecting the mutation annotations to the protein domains indicates

the number of samples with the mutation. The most recurrent mutations (aa1450 and aa876) are labeled.

(B) Schematic diagram of (A) shows species of specific mutations. The truncated mutations accounted for 98.5 percent of the total mutants.

(C) Confocal images of live HEK293T cells expressing Axin (red) and APC ^{R1450*} (left, green) or APC ^{R876*} (right, green) puncta.

(D) Representative images of live CRC cells expressing species of pScarlet-tagged Axin mutant protein and APC^{R1450*} or APC ^{R876*} puncta.

(E) Representative images of live CRC cells expressing mcherry-tagged β-catenin and APC^{R1450*} (up) or APC ^{R876*} (down) when knockdown Axin with short hairpin RNA.

(F) Comparison of protein puncta numbers in SW480 cells expressing Axin short hairpin RNA. Horizontal lines indicate mean ± SEM.

Fig S6

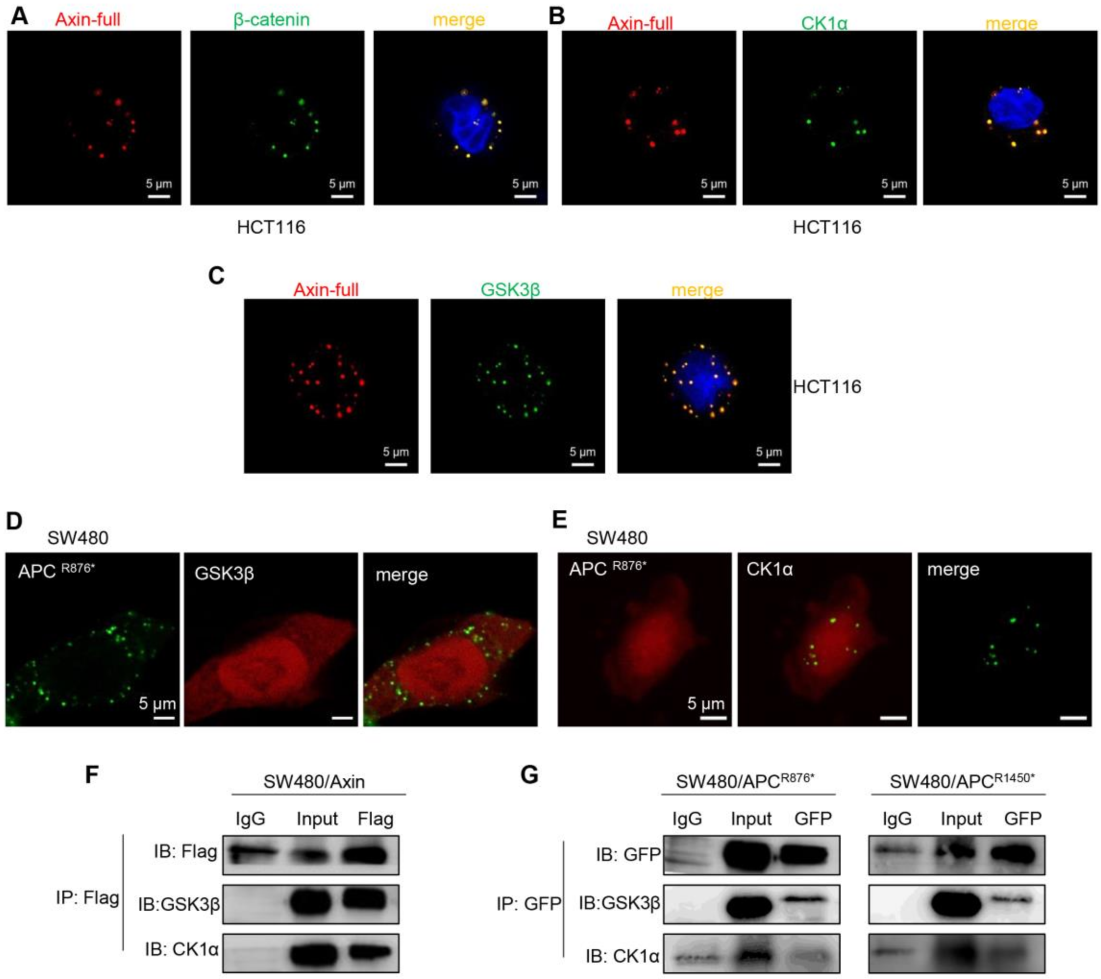

Figure S6

(A-C) Representative images of live HCT116 cells expressing Axin (red) and β-catenin(green) (A) or GSK3β(green) (B) or CK1α(green) (C) puncta.

(D-E) Representative images of live SW480 cells expressing APC^{R876*} (green) and GSK3β (red) (D) or CK1α (red) (E) proteins.

(F-G) Axin or GFP interacts with GSK3β or CK1α respectively in SW480 cells via Co-IP assay. Co-IP was performed using anti-Flag antibody (F) or anti-GFP antibody (G). IB, immunoblotting.

Fig S7

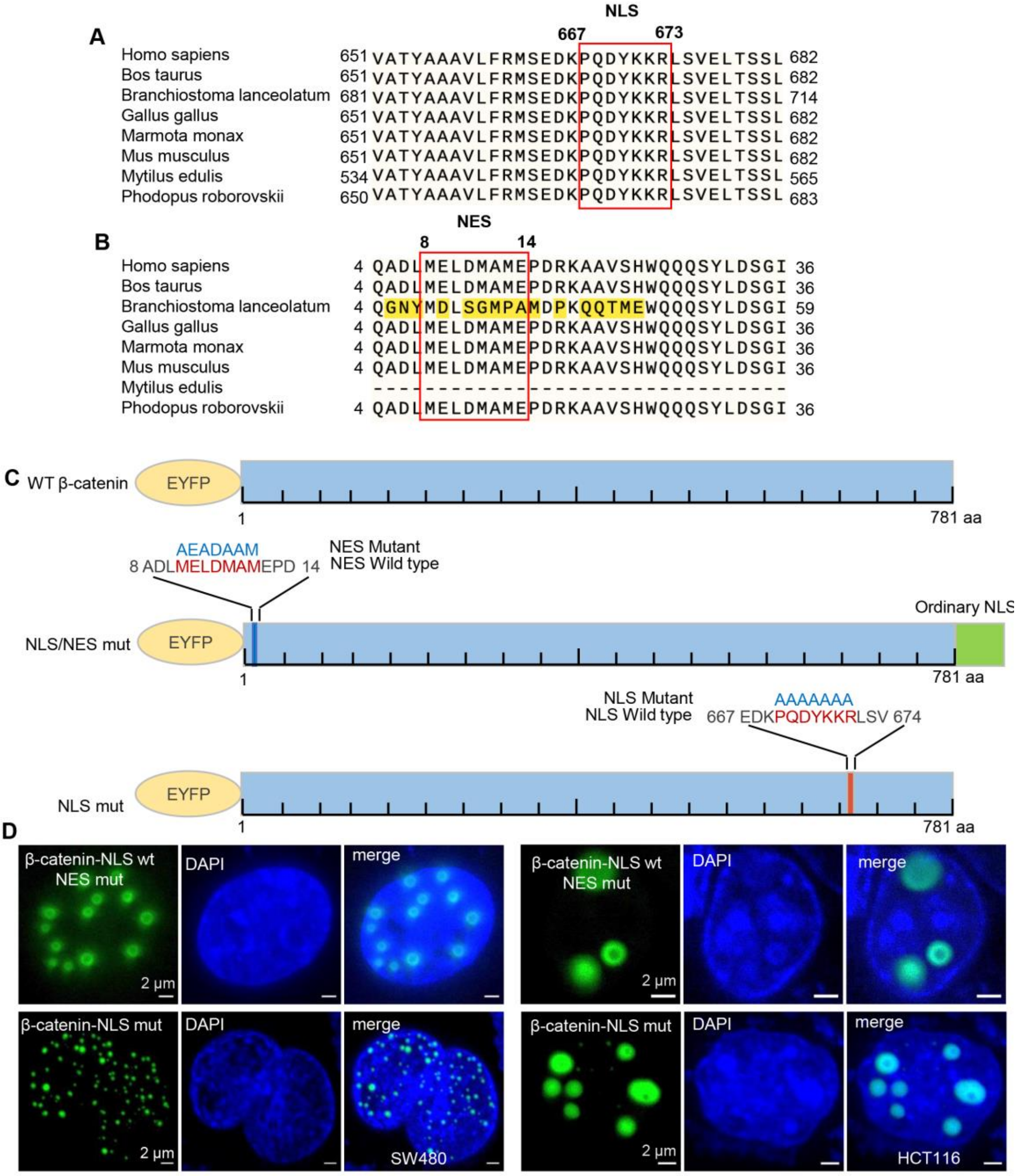

Figure S7

(A-B) The NLS (A) and NES (B) amino acid sequence of β-catenin were predicted by PSORT II.

(C) Schematic diagram of NLS-mutated or NES-mutated β-catenin derivatives tagged EYFP. Ectogenic NLS, predicated NLS and NES are indicated by the green box, red box and blue box, respectively. NLS, nuclear localization signals; NES, nuclear export signals; WT, wild type.

(D) Representative images of NLS wild-type but NES-mutated (up) or NLS-mutated (down) β-catenin derivatives in SW480 and HCT116.

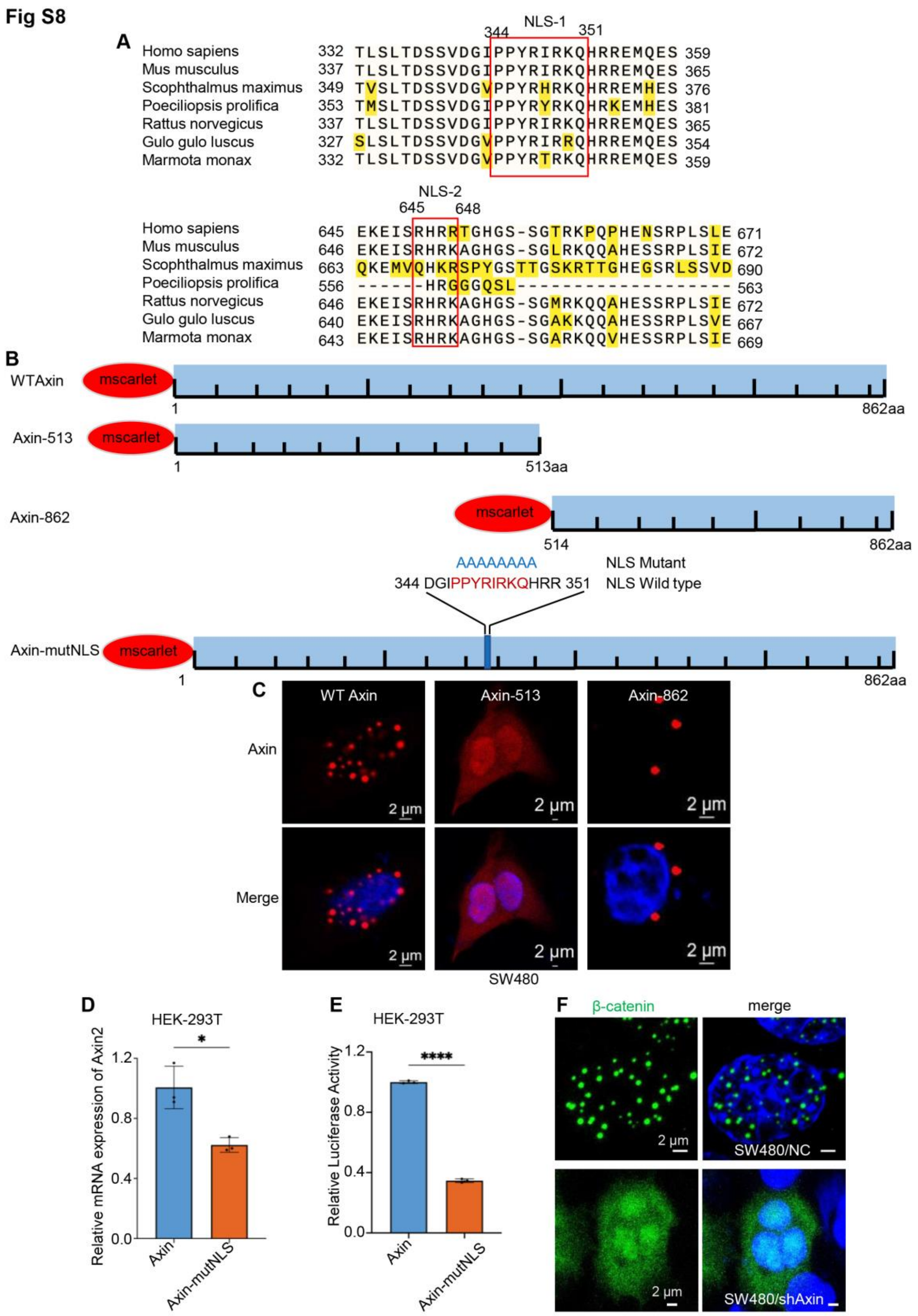

Figure S8

(A) The NLSamino acid sequence of Axin was predicted by PSORT II.

(B) Schematic diagram of NLS-mutated or truncated Axin derivatives tagged pScarlet. Predicated NLS

is indicated by blue box. NLS, nuclear localization signals; WT, wild type.

(C) Representative images of Axin (red) and nuclear (DAPI, blue) expression after transfection of truncated Axin (Axin-513, Axin-862) in SW480.

(D) The mRNA levels of Wnt pathway-specific downstream target gene (Axin2) was examined by RT–PCR in HEK293T cells expressing wild-type Axin or NLS-mutated Axin. Error bars, mean ± SD; *, p <0.05.

(E) Analysis of TOP/FOP reporter activity in HEK293T cells transfected with wild-type Axin or NLS-mutated Axin. Error bars, mean ± SD; ****, p<0.0001.

(F) Representative images of β-catenin (green) and nuclear (DAPI, blue) in SW480 (up) and HEK293T (down) cells expressing control vector and Axin short hairpin RNA.

Reference

1. J. Nong, K. Kang, Q. Shi et al (2021) Phase separation of Axin organizes the β-catenin destruction complex. J Cell Biol 220:
